# A Courageous Journey of a Foreign-Born Intensive Care Unit Nurse in Finland: A Narrative Case Study

**DOI:** 10.1177/15271544241309312

**Published:** 2025-01-29

**Authors:** Floro Cubelo

**Affiliations:** 1 School of Wellbeing and Culture, Healthcare Sector, 281672 Oulu University of Applied Sciences, Oulu, Finland; 2 International Coordination & Management Affairs, The Filipino Nurses Association in the Nordic Region, Oulu, Finland; 3 Department of Nursing Science, Faculty of Health Sciences, University of Eastern Finland, Kuopio, Finland

**Keywords:** policy, migrant, international nurse, intensive care unit, workforce, narrative

## Abstract

Finland is facing a severe shortage of nurses. While uncommon, the deportation of a foreign-born nurse could exacerbate this already critical situation. However, research on the deportation experiences of migrants, particularly healthcare workers such as nurses, remains scarce. This study aimed to provide a descriptive analysis of a deportation case involving a foreign-born intensive care nurse in Finland and examined the implications of this case for nursing education, the healthcare workforce, and government policies. Using a narrative case study approach, publicly available data from various sources, including mainstream and social media platforms, were analyzed. The nurse in this narrative left the country following disappointment with government officials, despite winning the deportation case. The findings of the study also revealed that the deportation decision faced by the foreign-born intensive care nurse has significant implications for nursing education, healthcare management, and government policies, indicating the need for necessary reforms. Intergovernmental collaboration is crucial to expedite the just and equitable processing of residence permits for highly skilled migrant health workers, promoting more effective government policies.

## Introduction

Finland faces a significant shortage of nurses, with a monthly demand of approximately 4,300 registered nurses in 2022 (Ministry of Economic Affairs and Employment Services, 2022). To address this shortage, Finland recruits internationally educated nurses from countries with fragile healthcare systems, such as the Philippines (Cubelo et al., 2024). Between 2006 and 2017, 37% of nurses who migrated to Finland were foreigners (neither Finnish citizens nor those born in Finland). In 2023, 73,236 foreigners migrated to Finland, compared with 49,998 in 2022, a 46% increase. This number has fluctuated over the years. Nearly half (47%) of these foreign nurses came from Estonia. Finland may attract Estonian nurses for economic reasons, geographical proximity, and possible language skills. Following Estonia, Finland has attracted the most nurses from Russia, the Philippines, Spain, China, and Sweden (Juuti, 2020). The country will recruit more nurses from India, the Philippines, Brazil and Vietnam (Finnish Government, 2023).

As the demand for foreign workers grows, the number of applicants at the Finnish Immigration Service (Migri) increases correspondingly. In several other countries, such as Canada, New Zealand, and the United Kingdom, immigration decisions are influenced by labor demands and the potential economic contributions of the applicants (Gov, 2020; Government of Canada, 2022; New Zealand Immigration, 2022). In Finland, the processing of applications depends on the type of residence permit and the time required for investigation (Finnish Immigration Service, n.d.-b). For nurses, the residence permit is work-based and initially classified as Type A, signifying a continuous residence permit for those intending a long-term stay. After four years of uninterrupted residence with a Type A permit, nurses are eligible to apply for a permanent residence permit (Type P) (Finnish Immigration Services, n.d.-a).

Despite the demand for migrant workers, negative decisions are still common. In 2022, out of 12,496 decisions made by Migri regarding work permits for first-time applicants, 2,296 (18.5%) were negative. This represents a 37% increase compared with the previous year, when 1,439 negative decisions were made (Finnish Immigration Service, 2023).

The literature on the deportation experiences of migrants, particularly healthcare workers, is sparse. Data on the deportation of healthcare professionals in Finland are unavailable, and the deportation of foreign-born nurses is uncommon. However, in late 2022, Helsingin Sanomat reported a significant case of a foreign-born nurse of Asian descent who received a deportation decision from Migri (Kuokkanen, 2022; Palkoaho, 2022). Studies have shown a relationship between structural racism and the enforcement of immigration law (Aranda & Vaquera, 2015; Provine, 2013). Experts believe that racism within the criminal justice system can lead to consequences for deportation (de Noronha, 2019).

Moreover, there is a specific gap in research regarding healthcare professionals’ deportation experiences and the fairness of the process. A scientific inquiry into the recent deportation case of a foreign-born nurse, including the background and adjudication process, is needed to address its implications for the nursing profession and policy reforms to retain skilled migrant nurses. This case also has potential global implications, especially for developed countries recruiting foreign-born nurses and international nursing students to address nursing shortages.

## Objective

**Figure 1.**
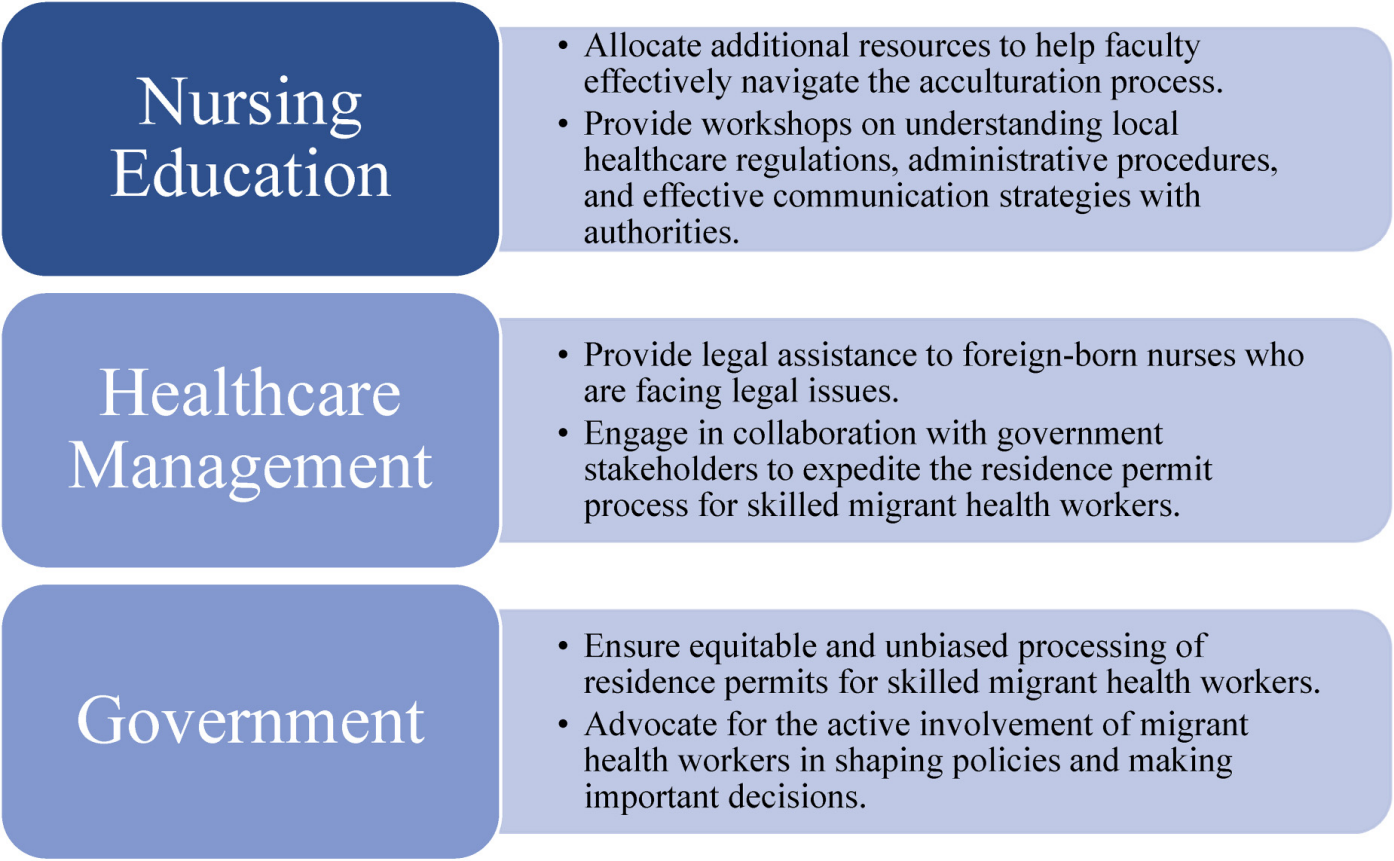
Policy suggestions to enhance the support and empowerment of nurses with foreign backgrounds in Finland.

The objective of this study was to provide a descriptive analysis of deportation cases involving a foreign-born ICU nurse who graduated in Finland. The study seeks to examine the implications of this case for nursing education, the healthcare workforce, and government policies (Figure 1). By exploring this specific case, a comprehensive understanding of the potential impact on these key areas can be gained.

## Methodology

### Study Design

This study employed a narrative case study approach (Table 1). According to Thyer (2010, Chapter 20), the narrative case study methodology enables researchers to analyze intricate and complex cases comprehensively, facilitating a detailed understanding with rich contextual details. It allows for the integration of past and present elements, providing a solid foundation for further investigation (Thyer, 2010, Chapter 20). This approach is particularly valuable when studying unusual or rare situations that involve complex dynamics and multiple variables, which may be less suitable for other research methods (Thyer, 2010, Chapter 20).

**Table 1.** Structure of a Narrative Case Study of the Expatriation Case of a Foreign-Born Intensive Care Unit Nurse.

Brandell and Varkas structure of a narrative case study (Thyer, 2010, Chapter 20).	Information
Identify the issue	Expatriation of a foreign-born intensive care unit nurse
Finding literature	Use of academic databases including CINAHL, Scopus, Pubmed, and Pro-quest regarding the expatriation of migrants
Identify methods for data collection	Mainstream and social media- newspaper, online news, social media platforms
Describing analyzed data	Cross-referencing of public legal data available online by the Finnish government regarding the violations in question to ensure objectivity in the analysis of the issue
Conclusions	Implications of the study in nursing education, the healthcare workforce, and government policies were stated

### Data Collection

This narrative case study, conducted from December 2022 to March 2023, utilized publicly available data from various sources. These sources included mainstream media and social media platforms associated with reliable news outlets in Finland, presenting information in both Finnish and English languages.

### Data Analysis

This study adhered to the narrative case study framework outlined by Thyer (2010, Chapter 20). Simultaneously, the author cross-referenced public legal data available online by the Finnish government regarding the violations in question to ensure objectivity in the analysis of the issue. The study focused on the expatriation case of a foreign-born ICU nurse and sought to provide a balanced presentation by considering and incorporating perspectives from both sides of the situation.

### Trustworthiness and Rigor

In this narrative study, adherence to the principles of verisimilitude and utility (Loh, 2015) was maintained. Verisimilitude was ensured by portraying the study as accurate, believable, resonant, and plausible to the consumers (Blumenfeld-Jones, 1995; Loh, 2015). In terms of utility, the study was designed to be beneficial for future research, further investigation, and for the educational community, enhancing its practical value and applicability (Loh, 2015).

### Ethics Permit

The participant being studied has voluntarily revealed her identity in public and on various mainstream and social media platforms. Expert consultations were carried out, and it was concluded that ethics approval was not needed for this research because all the information was in the public domain. The nurse's actual name was not disclosed and instead was given a pseudonymous name, although it was possible to deduce the actual name.

## Results

### Facts of the Case

Nurse X migrated to Finland in 2015 to pursue a degree program in nursing (DPN) conducted in English. During her studies, she obtained the necessary credits to work as a nursing student in both the Home Care Services Unit of the City of Helsinki and Helsinki University Hospital (HUS). In Finland, nursing students are allowed to work once they have earned the required school credits on the basis of job complexity and associated duties. Nurse X had multiple short-term contracts throughout her employment (Kuokkanen, 2022).

Prior to the expiration of her residence permit, Nurse X applied to Migri for the renewal of her residence permit for work reasons. However, in December 2020, she received a negative decision from the agency, resulting in her deportation order and a two-year ban on entry into the Schengen area. The Schengen area, which includes Finland, allows residents to travel freely within most European Union member states with few restrictions. It includes the majority of European Union member states, with the exceptions of Cyprus and Ireland (European Commission, 2024). Migri provided a general description of alleged submissions of falsified documents but did not specify the specific claims of refusal, such as whether it pertained to the bank account, payslips, or job contracts as evidence of income. The agency justified the lack of disclosure by citing the “public interest” (Kuokkanen, 2022).

Nurse X openly shared her dissatisfaction with Migri and her treatment by the agency in a video posted on her social media account. During the interrogations, Migri accused her of submitting account statements that allegedly contained numerous miscalculations, raising doubts about their authenticity.

Hi! My name is xxx. I am going to tell you a little bit about myself. I am originally from Mongolia. I moved to Finland five years ago to study nursing and graduated two years ago. I am working in the Intensive Care Unit taking good care of COVID-19 patients, but I received a deportation decision from the Finnish Immigration Services. The grounds of the decision were concealed from me because it would go against a “very important public interest”. For five months, I didn’t really know what led to this decision that I could defend myself. In the end, I received information from the Police to invite me for a hearing because Migri accused me of account statements that were counterfeited. How is it even possible nowadays to falsify a bank document in Finland?

According to Nurse X, there was a significant delay in the decision-making process by the justice system, and she felt that her fundamental human rights were violated. Deportation is a critical decision made by a group of influential authorities, and individuals who are unable to publicly share their experiences may struggle to have their voices heard by the government. Despite the ruling, Nurse X was permitted to continue working while awaiting the outcome of the appeal.

However, the judicial system is so slow that it almost took two years before I was found innocent, and the administrative court annulled Migri's decision. For the past two years, I have been living in Finland without my fundamental rights- leave the country, meet my family, and receive a bank account. But the saddest part is that after this whole battle, in the best-case scenario, the Migri received a reprimand from the head of the parliamentary judiciary. Just a reprimand. And that's it. There are structural problems in the Immigration Services because this is not the fault or incompetence of one person. The deportation decision is always made by a big team of…senior officials. And you already know from the news about Migri's terrible decision. And think of those whose stories don't make the news.

### Deportation, Trial, and Migri's Side

Under the Aliens Act Section 149 (565/2019), deportation (the removal of an alien from the country) is based on the following grounds (Finlex, 2020):

- residing in the country without a valid residence permit;
- sentenced guilty with a year or more maximum imprisonment/guilty of repeated offenses;
- considered a public threat; and
- suspicion of activities endangering Finland's national security.

Migri did not disclose the specific grounds of the negative decision, citing it as contrary to an important public interest. However, the Helsinki Times news outlet revealed the following grounds on which the decision was based (Kuokkanen, 2022):

- presented forged documents on several occasions;
- falsification related to the declaration of subsistence;
- suspicion of bank account fraudulence;
- employment relationships were of short duration; and
- The applicant's employment relationships cannot be particularly stable and permanent.

According to the police interrogation transcript dated May 27, 2021, the bank statement provided by Nurse X contained numerous calculation errors that would not be present in a genuine bank account (Kuokkanen, 2022). If the decision made by Migri was based on the use of forged documents, this act is punishable under Chapter 33 (769/1990), Section 1 of the Criminal Code, which states (Finlex, 2022):

A person who prepares a false document or other exhibit or falsifies one for it to be used as misleading evidence or uses a false or falsified exhibit as misleading evidence shall be sentenced for forgery to a fine or imprisonment for at most two years.

Following Migri's decision in 2020, the appeal case underwent a two-year process before receiving a verdict. On November 1, 2022, the Helsinki Administrative Court overturned the negative decision and returned the case to Migri. Additionally, the court criticized the agency for rejecting the application on the basis solely of suspicion and failing to provide reasons for its decision, which is in violation of the law (Kuokkanen, 2022).

By the end of 2022, the newly appointed director general of Migri, Ilkka Haahtela, expressed a sincere apology to Nurse X and the entire country (Jaakkonen, 2022). In a press release, the government agency acknowledged that mistakes were made in interpreting the law and handling the case. The Act on the Openness of Government Activities was not properly followed, and Nurse X's right to appeal was not adequately considered in the assessment of confidentiality. To address these issues, the agency plans to commission an external audit and implement reforms on the basis of its findings. The goal is to improve procedures and instructions, ensuring that the client's situation is more comprehensively taken into account when interpreting the law (Finnish Immigration Service, 2022).

## Discussion

The case of the deportation of the foreign-born nurse is open to interpretation from both political and legal perspectives. According to Veronika Honkasalo, a local politician in Finland, the incident exemplified the underlying racism prevalent in Finnish culture, where immigrants are initially viewed with suspicion and where concerted efforts are made to substantiate those suspicions (Honkasalo, 2022). This assertion aligns with the findings of a 2019 study by the European Union Agency for Fundamental Rights, which revealed that 63% of individuals of African heritage in Finland had encountered racially motivated harassment, exceeding the average of 30% across the 12 European Union countries examined. This proportion was also 41% in Denmark and Sweden (Kataja, 2020). Additionally, racism toward Asians intensified during the COVID-19 pandemic in Finland (Koivisto & Hirvonen, 2020). The issue of racism is not novel in Finland but rather deeply ingrained and pervasive in contemporary society (Stenroos, 2023).

Richard and Brisbois (2019) highlight that detention and deportation have wide-ranging implications, affecting ethics, society, humanitarianism, law, finances, health, and politics. These impacts include feelings of injustice after long-term residence and employment, a lack of support networks, limited legal representation, stigmatization, and inadequate healthcare resources in the home country.

As nursing education becomes increasingly multicultural, it is crucial for nursing faculty to understand the challenges of acculturation and provide appropriate support and resources. Nurse X's case underscores the importance of allocating additional resources to help faculty effectively navigate the acculturation process. Furthermore, to better prepare future nurses of foreign backgrounds for bureaucratic challenges with authorities, faculty should integrate specific training and guidance in navigating these systems. This could involve workshops on understanding local healthcare regulations, administrative procedures, and effective communication strategies with authorities. By doing so, nursing faculty can empower their students to handle bureaucratic challenges confidently and succeed in their professional roles.

Finland currently faces a critical shortage of nurses, especially in specialized units, necessitating measures to retain skilled migrant healthcare professionals. Foreign-born nurses educated and trained in Finland should be prioritized for retention. This requires strengthening retention strategies and revising migration policies to support healthcare professionals who wish to remain in the country. The case of Nurse X can potentially discourage migrant nurses from choosing Finland, exacerbating the nursing shortage. Therefore, employers, particularly in the public sector, should provide comprehensive legal services and support to employees, extending beyond workplace and patient safety concerns. This approach can prevent the loss of competent workers who have been trained in Finland, ensuring the stability and quality of the healthcare workforce.

Higher management levels in hospitals and healthcare organizations must also play a role in supporting the retention of foreign-born nurses. They should advocate for and show support to nurses of diverse backgrounds, recognizing their valuable contributions to the healthcare system. This can be achieved through the provision of resources, mentorship programs, cultural orientation, and opportunities for career development (International Council of Nurses, 2019). Creating a supportive and inclusive work environment communicates the value placed on nurses of all backgrounds and can foster their continued commitment to the organization.

Furthermore, healthcare management should acknowledge the importance of legal services in assisting healthcare professionals facing immigration-related challenges. It is imperative for healthcare institutions employing nurses of foreign backgrounds to make decisions that facilitate the promotion of educational aspects among all professionals, aiming to provide proficient, expedient, and efficient responses to their service requirements (Rodríguez et al., 2014). Access to legal support, either through internal resources or external partnerships, can help nurses navigate complex immigration processes and address any legal issues they may encounter. By providing this support, organizations demonstrate their commitment to the well-being and professional development of all employees, irrespective of their background.

Collaboration with relevant stakeholders (Cubelo et al., 2024), such as immigration authorities and professional associations, is also vital. Nursing management should work together with these entities to advocate for policies that support the retention of foreign-born nurses. This may involve addressing systemic issues in immigration processes, advocating for fairness and transparency in decision-making, and promoting diversity and inclusivity within the nursing workforce. Through collaborative efforts, a supportive environment can be created that attracts and retains skilled nursing professionals.

In terms of government policy reforms, Finland should prioritize the efficient processing of residence permits for skilled migrant nurses, particularly in areas with a high degree of nursing shortage. Streamlining the immigration process and providing timely approval of permits can attract and retain foreign-born nurses who can help address the shortage. The issue of systemic racism, as highlighted by a policymaker, requires a comprehensive investigation by the government. Taking appropriate actions to eliminate biases and discriminatory practices within social and healthcare systems is crucial. This may involve implementing diversity and inclusion initiatives, providing cultural sensitivity training (Červený et al., 2022; Merry et al., 2021), and promoting equal opportunities for healthcare professionals of all backgrounds.

Following fundamental human rights, individuals accused of a crime, such as submitting falsified documents, should be granted the opportunity to defend themselves adequately (Finlex, 2022). It is important for the government, represented by institutions such as Migri, to ensure that decisions are fair, just, and transparent.

Nurses recruited from EU countries, such as Spain, often leave Finland shortly after their arrival because of the cold weather, high cost of living, and difficulties in learning the Finnish language (Sulenko, 2016). Nurse X, who had made significant efforts to integrate into Finnish society by attaining fluency in the language, eventually left the country to go back to Mongolia following disappointment with government officials, despite winning the deportation case. Nurse X expressed a willingness to advocate and raise awareness for newcomers to Finland, highlighting the challenges faced by foreign-born individuals in their efforts to establish themselves within the healthcare system and society at large.

## Study Limitations

This narrative case study has limitations and requires further evidence for broader relevance and specific impacts on foreign nurses. While significant, its findings are not generalizable to all foreign-born nurses. However, case studies provide new scientific developments (Flyvbjerg, 2006) for future research. Placing this case within the context of negative residence permit decisions could clarify its implications. Additional research is needed to assess the impact on nursing education, management, and policies, especially regarding challenges faced by migrant healthcare workers due to denied permits.

## Conclusion

The issue of deportation carried significant weight, as it involved crucial decisions made by higher authorities within the government. Given Finland's pressing nursing shortage, the country must retain foreign individuals who have received specialized education and training within its borders. Particularly during the COVID-19 pandemic, Finland actively sought nurses from non-EU countries to address the staffing shortfall in nursing homes and secondary-level healthcare facilities. With an anticipated increase in reliance on foreign labor in the coming decades, formulating policies for migrant workers should be a central topic of discussion at the parliamentary level. This study further highlighted that even in a country such as Finland, which possesses a functional judicial system, there can be lapses in the processing of serious allegations of legal violations. Highly skilled migrant or foreign-born healthcare professionals have the potential to address the growing concerns surrounding healthcare staffing shortages in the country.

## References

[bibr1-15271544241309312] Aranda E. Vaquera E. (2015). Racism, the immigration enforcement regime, and the implications for racial inequality in the lives of undocumented young adults. Sociology of Race and Ethnicity, 1(1), 88–104. 10.1177/2332649214551097

[bibr2-15271544241309312] Blumenfeld-Jones D. (1995). Fidelity as a criterion for practicing and evaluating narrative inquiry. International Journal of Qualitative Studies in Education: QSE, 8(1), 25–35. 10.1080/0951839950080104

[bibr3-15271544241309312] Červený M. Kratochvílová I. Hellerová V. Tóthová V. (2022). Methods of increasing cultural competence in nurses working in clinical practice: A scoping review of literature 2011–2021. Frontiers in Psychology, 13, 936181. 10.3389/fpsyg.2022.936181 36092120 PMC9449514

[bibr5-15271544241309312] Cubelo F. Turunen H. Jokiniemi K. (2024). Recruit, integrate, and retain: Internationally educated nurses mobility to the Nordic region: A two-round policy Delphi study. Nursing Outlook, 72(6), 102299. 10.1016/j.outlook.2024.102299 39500071

[bibr6-15271544241309312] de Noronha L. (2019). Deportation, racism and multi-status Britain: immigration control and the production of race in the present. Ethnic and Racial Studies, 42(14), 2413–2430. 10.1080/01419870.2019.1585559

[bibr7-15271544241309312] European Commission. (2024). Schengen Area. Migration and Home Affairs. https://home-affairs.ec.europa.eu/policies/schengen-borders-and-visa/schengen-area_en

[bibr8-15271544241309312] Finlex. (2020). Aliens Act. Finlex. https://www.finlex.fi/en/laki/kaannokset/2004/20040301

[bibr9-15271544241309312] Finlex. (2022). Criminal Code. Finlex. https://www.finlex.fi/en/laki/kaannokset/1889/en18890039

[bibr10a-15271544241309312] Finnish Government. (2023). A strong and committed Finland: Programme of Prime Minister Petteri Orpo’s Government. Urn.Fi.. https://urn.fi/URN:ISBN:978-952-383-763-8

[bibr10-15271544241309312] Finnish Immigration Service. (2022). Finnish Immigration Service conducts inquiry into case of Mongolian nurse. Maahanmuuttovirasto. https://migri.fi/en/-/finnish-immigration-service-conducts-inquiry-into-case-of-mongolian-nurse

[bibr11-15271544241309312] Finnish Immigration Service. (2023). Statistics. Migri.Fi. https://statistics.migri.fi/#decisions/21205/59/2/105?start=612&end=623

[bibr12a-15271544241309312] Finnish Immigration Service. (n.d.-a). Residence permit types. Maahanmuuttovirasto. Retrieved December 27, 2024, from https://migri.fi/en/residence-permit-types

[bibr12-15271544241309312] Finnish Immigration Service. (n.d.-b). What affects the processing time of your application? Maahanmuuttovirasto. Retrieved February 11, 2023, from https://migri.fi/en/expected-processing-time-what-affects

[bibr13-15271544241309312] Flyvbjerg B. (2006). Five misunderstandings about case-study research. Qualitative Inquiry: QI, 12(2), 219–245. 10.1177/1077800405284363

[bibr14-15271544241309312] Gov U. K. (2020). The UK’s points-based immigration system: policy statement. *Gov.uk*. https://www.gov.uk/government/publications/the-uks-points-based-immigration-system-policy-statement/the-uks-points-based-immigration-system-policy-statement

[bibr15-15271544241309312] Government of Canada. (2022). Priority processing for temporary workers in essential occupations. *Canada.Ca*. https://www.canada.ca/en/immigration-refugees-citizenship/corporate/publications-manuals/operational-bulletins-manuals/service-delivery/coronavirus/temporary-residence/work-permit.html

[bibr16-15271544241309312] Honkasalo V. [veronikahonka] (2022). Tweet on the case of Nurse xxx *Twitter*. https://twitter.com/veronikahonka/status/1601861356877221889?cxt=HHwWgsC8wdia-rosAAAA

[bibr17-15271544241309312] International Council of Nurses. (2019). International career mobility and ethical nurse recruitment. *Icn.Ch*. https://www.icn.ch/system/files/documents/2019-11/PS_C_International%20career%20mobility%20and%20ethical%20nurse%20recruitment_En.pdf

[bibr18-15271544241309312] Jaakkonen L. (2022). Koronateholla taistellut mongolialaishoitaja sai karkotuspäätöksen Suomesta – Migri ei ymmärtänyt tiliotetta ja syytti väärennyksestä: “Pahin vaihe elämässäni.” (A Mongolian nurse who fought with corona power was deported from Finland - Migri did not understand the bank statement and accused her of forgery: “The worst phase of my life”) *MTV Uutiset*. https://www.mtvuutiset.fi/artikkeli/hoitaja-kertoo-mtv-lle-jarkytyksesta-kun-kuuli-tulleensa-karkotetuksi-alkoi-kahden-vuoden-piina-joka-kerta-kun-nain-poliisin-sydameni-tykytti/8591460

[bibr19-15271544241309312] Juuti M. . (2020). Sairaanhoitajien maastamuutto kasvussa ennen koronaa – muuttaneiden vuosittaiset määrät silti melko pieniä (Emigration of nurses on the rise before COVID19 pandemic - annual numbers of emigrants still relatively small). *Stat.fi*. https://stat.fi/tietotrendit/artikkelit/2020/sairaanhoitajien-maastamuutto-kasvussa-ennen-koronaa-muuttaneidenvuosittaiset-maarat-silti-melko-pienia.

[bibr20-15271544241309312] Kataja R. (2020). The happiest and the most racist: Institutional racism in Nordic countries. Harvard Political Review. https://harvardpolitics.com/nordic-racism/

[bibr21-15271544241309312] Koivisto J. Hirvonen S . (2020). Aasialaistaustaiset kokeneet Suomessa syrjintää koronavirusepidemian puhjettua: haukkumista viruksiksi, välttelyä ja epäasiallisia katseita (People of Asian origin have experienced discrimination in Finland since the outbreak of the coronavirus epidemic: being called viruses, shunned and inappropriate looks). *Yle Uutiset*. https://yle.fi/a/3-11190157

[bibr22-15271544241309312] Kuokkanen K. (2022). Työtaistelu (Industrial Action). Helsingin Sanomat. https://www.hs.fi/kaupunki/art-2000009149382.html

[bibr23-15271544241309312] Loh J. (2015). Inquiry into issues of trustworthiness and quality in narrative studies: A perspective. The Qualitative Report. 10.46743/2160-3715/2013.1477

[bibr24-15271544241309312] Merry L. Vissandjée B. Verville-Provencher K. (2021). Challenges, coping responses and supportive interventions for international and migrant students in academic nursing programs in major host countries: A scoping review with a gender lens. BMC Nursing, 20(1), 174. 10.1186/s12912-021-00678-0 34537039 PMC8449499

[bibr25-15271544241309312] Ministry of Economic Affairs and Employment in Finland. (2022). Occupational Barometer: Number of occupations suffering from labor shortage has risen to pre-COVID level. Työ- Ja Elinkeinoministeriö. https://tem.fi/en/-/occupational-barometer-number-of-occupations-suffering-from-labor-shortage-has-risen-to-precovid-level

[bibr26-15271544241309312] New Zealand Immigration. (2022). Green List and other immigration changes. New Zealand Immigration. https://www.immigration.govt.nz/about-us/media-center/news-notifications/green-list-and-other-immigration-changes

[bibr27-15271544241309312] Palkoaho M. (2022). Mongolialainen hoitaja: “Tämä ei ole Suomen edun mukaista” – Tehyssä tapaus järkyttää muttei yllätä (Mongolian nurse: ‘This is not in Finland's interest’ - shocking but not surprising case at Tehy). *Helsingin Sanomat*. https://www.hs.fi/kaupunki/art-2000009258407.html

[bibr28-15271544241309312] Provine D. M. (2013). Institutional racism in enforcing immigration law. Norteamérica, 8, 31–53. 10.1016/s1870-3550(13)71782-8

[bibr29-15271544241309312] Richard P. J. Brisbois M. D. (2019). Deportation and health: Implications for nurses: Implications for nurses. Nursing, 49(6), 64–66. 10.1097/01.NURSE.0000558095.53840.e9 31124859

[bibr30-15271544241309312] Rodríguez G. Angélica-Muñoz L. Hoga L. A. K. (2014). Cultural experiences of immigrant nurses at two hospitals in Chile. Revista Latino-Americana de Enfermagem, 22(2), 187–196. 10.1590/0104-1169.2980.2401 26107824 PMC4292601

[bibr31-15271544241309312] Stenroos M. (2023). Part 4: About the history of racism in Finland - THL. Finnish Institute for Health and Welfare. https://thl.fi/en/web/migration-and-cultural-diversity/support-material/online-course-on-anti-racism-for-professionals/part-4-about-the-history-of-racism-in-finland

[bibr32-15271544241309312] Sulenko K. (2016). Training in the country of origin as part of the international recruitment process and labor mobility immigration. An example is the recruitment of Spanish nurses in Finland. Tampere University. https://trepo.tuni.fi/bitstream/handle/10024/99342/GRADU-1466154330.pdf?sequence=1

[bibr33-15271544241309312] Thyer B. (2010). The handbook of social work research methods (2nd ed). Sage Publications.

